# Meta-Analysis of the Effects of the Catechol-O-Methyltransferase Val158/108Met Polymorphism on Parkinson’s Disease Susceptibility and Cognitive Dysfunction

**DOI:** 10.3389/fgene.2019.00644

**Published:** 2019-07-12

**Authors:** Chuanxi Tang, Wei Wang, Mingyu Shi, Na Zhang, Xiaoyu Zhou, Xue Li, Chengcheng Ma, Gang Chen, Jie Xiang, Dianshuai Gao

**Affiliations:** ^1^Department of Neurobiology and Anatomy, Xuzhou Key Laboratory of Neurobiology, Xuzhou Medical University, Xuzhou, China; ^2^Medical Technology School, Xuzhou Medical University, Xuzhou, China; ^3^Department of Rehabilitation Medicine, The Affiliated Hospital of Xuzhou Medical University, Xuzhou, China; ^4^Department of Neurology, The Affiliated Hospital of Xuzhou Medical University, Xuzhou, China; ^5^School of Nursing, Xuzhou Medical University, Xuzhou, China

**Keywords:** catechol-O-methyltransferase, Parkinson’s disease, genetic association, cognitive dysfunction, meta-analysis

## Abstract

**Background:** There is a continued debate and inconsistent findings in previous literature about the relationship of catechol-O-methyltransferase (COMT) and Parkinson’s disease (PD) susceptibility as well as cognitive dysfunction. To substantiate this existing gap, we comprehensively examine COMT genotype effects on the development of PD and test the hypothesis that the Met158 allele of the COMT gene is associated with cognitive dysfunction by conducting a meta-analysis review.

**Methods:** PubMed/MEDLINE, Embase, Cochrane databases search (18/30/08) yielded 49 included studies. Data were extracted by two reviewers and included COMT genotype, publication year, diagnostic status, ancestry, the proportion of male participants, and whether genotype frequencies were consistent with Hardy–Weinberg equilibrium. Unadjusted odds ratios (ORs) were used to derive pooled estimates of PD risk overall and in subgroups defined by ethnicity, gender, and onset of disease. Moreover, the association of certain cognitive domains in PD and COMT gene type was explored. Meta-analyses were performed using random-effect models and *p* value–based methods. All statistical tests were two-sided. The present study was registered with PROSPERO (CRD42018087323).

**Results:** In the current studies, we found no association between COMT Val158/108Met polymorphism and PD susceptibility. However, the gender-stratified analyses revealed marginally significant effects in heterozygote model analyses in women (*P* = 0.053). In addition, stratification according to onset of PD also shows significant effects of COMT Val158/108Met polymorphism on late-onset population both in recessive (*P* = 0.017) and allelic (*P* = 0.017) genetic models. For the intelligence quotient (IQ) score and Unified Parkinson Disease Rating Scale III (UPDRS III), there was no evidence for genetic association, except in subgroup analyses in Asian populations (IQ score, *P* = 0.016; UPDRS III, *P* < 0.001).

**Conclusion:** The COMT Val158/108Met polymorphism is associated with the risk for PD in female or late-onset PD. Methionine/methionine carriers of Asian population performed significantly worse than the valine allele carriers in IQ score and UPDRS III.

## Introduction

Parkinson’s disease (PD) is a common neurodegenerative disorder that leads to a syndrome that is related to neurological control of movement as well as other brain functions including cognition (William, 2006). Due to the high prevalence and poor treatment, it is associated with an increasing socio-economic burden occupying a wide spectrum from minimal disability to marked impairment of capabilities with respect to independence, safety, and communication (Gómez-Esteban et al., 2007; LeWitt, 2008). The development of PD is mainly connected with aging. However, there have been other confirmed risk factors that influence the occurrence of PD.

First and foremost, protein pathological hypothesis displays complex and distinctive pathophysiological profiles: accumulation of aberrantly processed and misfolded proteins, such as amyloid- β (Aβ), tau, α-synuclein, TAR DNA-binding protein 43 (TDP43) (Dikic, 2017; Galluzzi et al., 2017), and mutant forms of Huntingtin (Htt) (Boland et al., 2018). The clearance of these proteins is impaired (Ciechanover and Kwon, 2017), which aggravates the pathological accumulation aberrantly and neurotoxic effects (Menzies et al., 2017). Next, oxidative stress and neuro-inflammation cause non-autonomous cell death due to complex multi-factors interaction, including α-synuclein accumulation, ubiquitin-proteasome system dysfunction, and metabolic disturbances (Duyckaerts et al., 2010), such as hyperammonemia (Choudhury and Borah, 2015). There is strong evidence that demonstrates that enteric nervous system is involved in PD pathological progression toward the central nervous system. Gut–brain axis suggests that PD starts in the enteric nervous system and spreads to olfactory bulb or brainstem, a process that antedates degeneration of the dopaminergic nigrostriatal system (Halliday et al., 2012; Goedert et al., 2013). Among the reasons for this are involved in interacting with the microenvironment [outer: microbiota (Mayer et al., 2015), metabolites, and nutrients (Holmqvist et al., 2014); inner: immune cells (Neunlist et al., 2014) and inflammatory cytokines] of the gut and specific living conditions (Gatto et al., 2009). In addition, the above several aspects are ascribed to the root of genetic susceptibility. Genome-wide association studies (GWAS) have identified numerous genetic variants associated with the highly penetrant autosomal dominant or recessive PD (Vila and Przedborski, 2004). Recent studies have shown that genetic variants can influence cognitive abilities. The ensuing molecular insights have contributed to substantial advances in our understanding of the pathogenesis of PD. Further mechanism studies have shown that NUS1 loss can reduce the number of dopaminergic neurons and the dopamine level, and induce apoptosis events in the brain of fruit flies (Guo et al., 2018).

Research on genetics, however, has always been hampered by inadequate sample size. Meanwhile, gene phenotypes might be better targets for exploring the etiology and pathophysiology of PD. Although the diagnosis of PD relies on the presence of motor deficits and clinical effects of dopamine therapy, this disease is closely related with non-motor symptoms and signs before the onset of the classical motor symptoms. Indeed, some evidence has suggested some of gene phenotypes are dominated by non-motor symptoms, such as cognitive impairment (Marras and Chaudhuri, 2016).

Catechol-O-methyltransferase (COMT) is involved in catecholamine degradation (Vidgren et al., 1994). The Val158/108Met single nucleotide polymorphism (SNP) in COMT gene has been extensively investigated in relation to PD, as well as the cognitive dysfunctions, including five aspects: executive function, attention and working memory, language, memory, and visuospatial function (Goetz et al., 2008). It contains a common functional SNP at codon 158/108 and generates a valine (Val)-to-methionine (Met) substitution (Val158Met), which results in a reduction of COMT enzyme activity and an increase in the dopamine level in the prefrontal cortex (Meyer-Lindenberg et al., 2005; Scheggia et al., 2018). The association between COMT genotype and cognition symptoms in subjects with PD has been checked, but the results are controversial. In their results, Barnett et al. (2008) did find a robust association between Val158Met and IQ. Healthy individuals with Val/Val have poorer scores on working memory (Aguilera et al., 2008). However, the other neurocognitive phenotype studies demonstrated substantial between-study heterogeneity. Dennis et al. reported that COMT val108/158met genotype has no effect on cognitive behavioral measures in healthy individuals but has an impact on neural activation patterns. Beyond that, Porter et al. (2019) found no previous associations between COMT Val158Met and cognitive performance in the cohort of cognitively normal older adults. In PD, many similar explorations focusing on genetic factors in cognitive decline were also made. COMT genotype was associated with attention but not with overall cognitive status (Bialecka et al., 2012; Morley et al., 2012). Certainly, some studies asserted that COMT Met/Met genotype could be a predictor of faster cognitive decline in PD (Paul et al., 2016).

To systematically synthesize these disparate studies, we attempted to synthesize the evidence regarding COMT Val158/​108Met polymorphism and occurrence of PD, so much as PD with cognitive disorder through meta-analytic techniques. Therefore, the present study aimed to further our understanding of the effect of the COMT Val158/108Met variant on cognition function of PD. It would also be useful to reveal the mixed findings concerning COMT and PD in GWAS correlational research.

## Methods and Materials

Systematic review and meta-analysis were performed using the preferred reporting items for systematic review and meta-analysis statement (PRISMA) (Moher et al., 2009). The protocol for the present study was registered with the PROSPERO registry (CRD42018087323).

### Search Strategy

We searched major scientific databases including but not limited to PubMed, EMBASE, the Cochrane Library, Web of Science, the WanFang databases, SinoMed databases, and the CNKI up to November 2018 with different combinations of the following words: “Parkinson Disease,” “Idiopathic Parkinson Disease,” “catechol-O-methyltransferase” or “COMT,” “rs4680,” and “cognitive,” “cognition,” “cognition disorders,” “perception,” “memory,” “executive,” “dementia,” and “attention.” The search was restricted to English and Chinese language publications. We also obtained additional articles using reference lists of articles identified in the initial searches.

### Inclusion and Exclusion Criteria

Qualiﬁed studies in this meta-analysis should satisfy the inclusion criteria:

1) case-control studies that assessed the relationship between COMT Val^158/108^Met polymorphism and PD susceptibility or reported the association between the COMT Val^158/108^Met and cognition in PD subjects regarding the association between COMT; 2) there were sufficient data of cases and controls to calculate an odd ratio (OR) with 95% conﬁdence interval (CI); 3) the genotypes distribution in the control group was consistent with Hardy–Weinberg equilibrium (HWE); 4) the study was published in the English or the Chinese language; 5) if there were numerous studies from the same population, only the latest one was entered.

Studies were excluded for the major following reasons: 1) no PD cognitive data reported; 2) sample comprised patients with 22q11 deletion syndrome (who have only one copy of the COMT gene); 3) studies of a different COMT polymorphism, or overlap of reported data between papers; 4) animal trials, review, and the studies that did not report the genotype frequencies were ruled out.

### Data Extraction

Data were independently extracted by two authors according to the inclusion and exclusion criteria listed above. Disagreements were resolved through discussion with a third author. The following information from included studies was extracted: ﬁrst author, published year, country, ethnicity, sample size, average age of sample, number of male and female participants, allele frequency distribution in case and control, sample size, mean, and standard deviation for each cognitive variable by genotype group. COMT genotypes were grouped according to the presence or absence of the Val allele (Val/Val or Val/Met vs. Met/Met). The Newcastle-Ottawa Scale (NOS) was used to assess the methodological quality of each study (Stang, 2010).

### Statistical Analyses

The HWE with an exact test was used to evaluate normal heterogeneity of the population. Six separate analyses as the allelic, recessive, dominant, homozygous, heterozygous, and additive genetic models were conducted in the present meta-analysis. Pooled odds ratios (ORs) with 95% conﬁdence intervals (95% CIs) were used to compare the relationship between COMT polymorphism and PD risk, and standardized mean differences (SMDs) with 95% CIs were calculated for continuous variables. The omnibus (Q) tests and I^2^ test were used to examine the heterogeneity among the studies. If P > 0.10 or I^2^ < 50%, the fixed-effect model was adopted. Otherwise, the random-effect model would be used. We made a prior assumption that the meta-analysis can be affected by study level variability determined by different inclusion criteria across RCTs, which is more appropriately addressed by a random-effects model over a fixed-effect model. The pooled OR was assessed by Z test and *P* < 0.05 level was considered as statistically significant. Sensitivity analysis was carried out by sequentially omitting one study at a time to estimate the stability of the result. Subgroup analysis was performed to assess the influence of potential moderators. Potential publication bias was estimated using Egger’s linear regression test by visual inspection of the funnel plot. All analyses were performed using Stata 12.0.

## Results

### Study Characteristics

**Figure 1:**
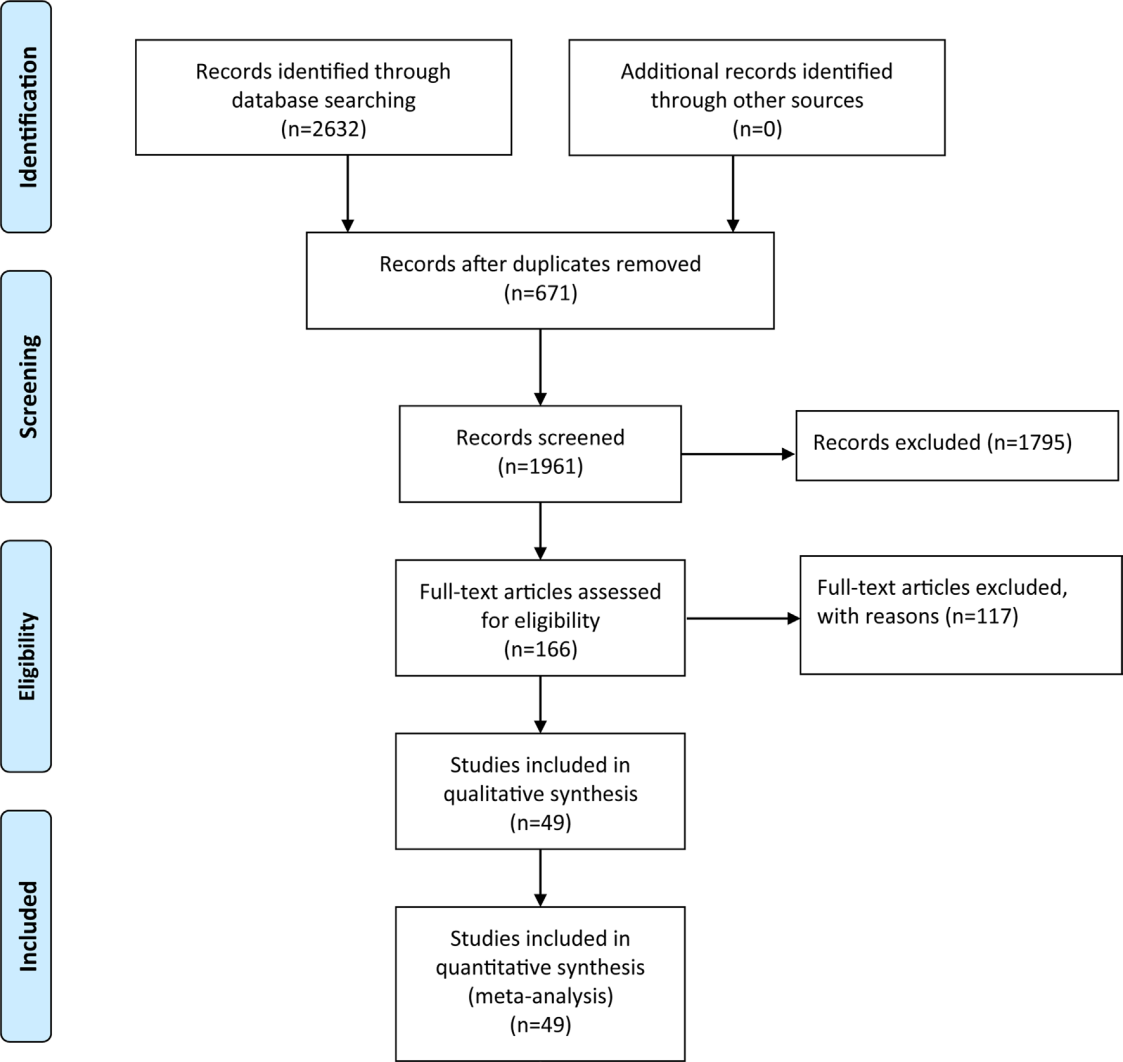
Flow diagram of articles selection process.

After the retrieval process, 49 relevant studies met the inclusion criteria, 35 (Hoda et al., 1996; Kunugi et al., 1997; Syvänen et al., 1997; Xie et al., 1997; Yoritaka et al., 1997; Mizuta et al., 2000; Lee et al., 2001; Wu et al., 2001; Eerola et al., 2002; Goudreau et al., 2002; Hernán et al., 2002; Xu et al., 2002; Lynch et al., 2003; Watanabe et al., 2003; Zhao et al., 2003; Shao et al., 2005; Bialecka et al., 2008; Kalinderi et al., 2008; Benitez et al., 2010; Rowe et al., 2010; Kiyohara et al., 2011; Bialecka et al., 2012; Torkaman-Boutorabi et al., 2012; Zeng, 2012; Klebe et al., 2013; Qi et al., 2013; Shih et al., 2013; Jin, 2014; Song et al., 2014; Wang, 2014; Song et al., 2015; Zhang et al., 2015; Paul et al., 2016; Qian et al., 2017; Ma et al., 2018) of which reported the association between COMT Val158/108Met polymorphism and the risk of PD, and a total of 11,773 patients and 17,046 controls were included in this section. Four (Bialecka et al., 2012; Wang, 2014; Dai et al., 2015; Li, 2016) studies reported the association between COMT Val^158/108^Met polymorphism and cognitive dysfunction in PD, whereas the other 10 (Williams-Gray et al., 2007; Williams-Gray et al., 2008; Hoogland et al., 2010; Morley et al., 2012; Wu et al., 2012; Fallon et al., 2015; Paul et al., 2016; Zhang et al., 2016; Xiao et al., 2017; Bäckström et al., 2018) articles mentioned the association between COMT Val158Met polymorphism and cognitive decline in PD including 1,547 PD patients. The selection process was shown in **Figure 1**, and the main characteristics of the included studies are listed in **Tables 1**–**3**.

**Table 1:** Characteristics of the included studies of the association between the COMT Val158Met polymorphism and PD susceptibility.

First author	Year	Country	Number of		Mean age		Male/female		Case			Controls				HWE		NOS score
			cases	control	cases	control	cases	control	V-V	V-M	M-M	V-V	V-M	M-M		cases	control	
Song et al.	2015	China	221	229	66.88	66.74	134/87	122/107	100	100	21	107	97	25		0.577	0.669	7
	Male		134	122	−	−	−	−	63	59	12	57	52	13		0.730	0.824	
	Female		87	107	−	−	−	−	37	41	9	50	45	12		0.631	0.698	
Zhang et al.	2015	China	437	530	62.4(11.4)	61.6(8.7)	230/207	318/212	251	173	13	291	210	29		0.009	0.262	6
Qi et al.	2013	China	90	95	63.2(10.3)	64.2(10.1)	47/43	50/45	59	26	5	45	34	16		0.356	0.040	6
	Early		44	51	−	−	−	−	28	14	2	24	18	9		0.883	0.0004	
	Late		46	43	−	−	−	−	31	12	3	21	16	7		0.246	0.206	
Jin et al.	2014	China	188	111	61.3(10.3)	58.3(9.7)	98/90	54/57	108	72	8	59	46	6		0.350	0.438	7
	Early		47	111	−	−	−	−	24	22	1	59	46	6		0.113	0.438	
	Late		141	111	−	−	−	−	84	50	7	59	46	6		0.900	0.438	
Ma et al.	2018	China	152	252	67.9(9.5)	67.8(10.8)	77/75	130/122	87	57	8	145	90	17		0.734	0.553	6
Wang et al.	2014	China	265	282	−	66.74(7.25)	−	141/141	143	107	15	150	117	15		0.386	0.199	7
Zhao et al.	2003	China	144	188	63.3(14)	55.8(15.5)	78/66	97/91	80	54	10	98	81	9		0.830	0.129	6
Song et al.	2014	China	237	247	−	−	146/91	133/114	109	107	21	118	102	27		0.466	0.486	6
	Late		199	196	−	−	−	−	95	84	20	91	21	84		0.821	0.001	
	Male		146	133	−	−	−	−	67	67	12	64	54	15		0.400	0.486	
	Female		91	114	−	−	−	−	42	40	9	54	48	12		0.907	0.784	
Zen et al.	2012	China	94	104	65.12(10.2)	67.06(10.28)	58/36	60/44	33	45	16	40	50	14		0.921	0.794	7
	Male		58	60	−	−	−	−	22	30	6	25	27	8		0.362	0.868	
	Female		36	44	−	−	−	−	11	15	10	15	23	6		0.319	0.546	
Xiao et al.	2017	China	143	157	65.1(8.7)	65.4(7.2)	79/64	88/69	79	56	8	91	57	9		0.637	0.985	7
	Early		24	157	−	−	−	−	8	15	1	91	57	9		0.073	0.985	
	Late		119	157	−	−	−	−	71	41	7	91	57	9		0.739	0.985	
Eerolaa et al.	2002	Finland	147	137	67.2	65.8	87/60	50/87	30	71	46	31	71	35		0.786	0.595	7
Watanabea et al.	2003	Japan	121	100	68.2	64(9)	49/72	85/15	57	47	14	47	49	4		0.377	0.043	7
Toraman-Boutorabi et al.	2012	Iran	108	70	57.46(10.35)	55.73(11.69)	72/31	42/28	20	50	33	19	32	19		0.892	0.473	7
	Male		72	50	−	−	−	−	13	38	21	12	19	11		0.560	0.539	
	Female		31	28	−	−	−	−	7	12	12	7	13	8		0.253	0.710	
Yoritaka et al.	1997	Japan	176	156	62.7(8.8)	57.9(16.2)	−	−	100	62	14	69	77	10		0.323	0.058	6
Benitez et al.	2010	Colombia	104	136	60.1(12.6)	62.4(9.5)	61/43	63/73	47	39	17	52	68	13		0.800	0.171	7
Xie et al.	1997	China	70	62	64.3(9.96)	63.9(9.86)	31/39	24/38	44	21	5	37	19	6		0.277	0.150	7
Syvanen et al.	1997	Finland	158	76	−	−	87/71	36/40	39	80	39	14	35	27		0.874	0.655	6
Kiyohara et al.	2011	Japan	238	369	68.5(1.1)	66.6(0.85)	91/147	140/228	98	116	24	179	166	24		0.222	0.076	6
Lee et al.	2001	Finland	73	49	62	−	33/40	−	40	27	6	26	21	2		0.636	0.371	6
Goudreau et al.	2002	USA	319	196	−	−	197/122	74/122	59	163	84	50	82	55		0.205	0.094	7
	Male		191	72	−	−	−	−	39	99	53	17	37	18		0.559	0.812	
	Female		115	115	−	−	−	−	20	64	31	33	45	37		0.186	0.203	
	Early		164	82	−	−	−	−	26	89	49	28	37	17		0.170	0.463	
	Late		142	105	−	−	−	−	33	74	35	22	45	38		0.613	0.209	
Kunugi et al.	1997	Japan	109	153	−	−	55/54	75/78	46	47	16	74	70	9		0.485	0.149	6
Hoda et al.	1996	UK	139	173	−	−	−	−	32	70	37	40	88	45		0.920	0.811	6
Klebe et al.	2013	France	5886	10723	57.6(13.8)	60.0(10.1)	3532/2354	5388/5335	1417	2911	1558	2526	5303	2894		0.429	0.313	8
Lynch et al.	2002	USA	100	66	60.6	46.5	71/29	30/36	29	45	25	20	30	16		0.374	0.477	7
Hernán et al.	2002	USA	213	439	−	−	−	−	50	106	57	112	220	107		0.958	0.960	6
	Male		101	203	−	−	−	−	26	44	31	50	102	51		0.203	0.944	
	Female		112	236	−	−	−	−	24	62	26	62	118	56		0.255	0.487	
Mizuta et al.	2000	Japan	171	199	66.0(8.0)	67.5(11.7)	−	−	85	71	15	88	90	21		0.975	0.776	7
Shih et al.	2013	China	260	100	71.4(0.787)	−	137/123	−	100	90	70	30	46	24		0.001	0.443	6
Kalinderi et al.	2008	Greece	134	125	61.7	71.7	−	−	48	54	32	40	49	36		0.035	0.016	7
Paul et al.	2016	USA	341	483	−	−	−	−	83	174	84	127	229	127		0.704	0.255	7
Bialecka et al.	2008	Poland	322	357	64.0(10.2)	72.5(9.7)	191/131	207/150	78	160	84	80	166	111		0.916	0.234	8
	Early		101	357	−	−	−	−	24	46	31	80	166	111		0.394	0.234	
	Late		221	357	−	−	−	−	54	114	53	80	166	111		0.637	0.234	
Białecka et al.	2012	Poland	57	64	−	−	−	−	38	15	4	38	23	3		0.167	0.839	6
Wu et al.	2001	China	222	191	67.2(9.1)	65.8(9.2)	162/62	145/52	125	79	18	117	62	12		0.277	0.336	6
	Early		37	34	−	−	−	−	25	16^a^		33	15^a^			−	−	
	Late		187	163	−	−	−	−	100	81^a^		86	57^a^			−	−	
	Male		160	140	−	−	−	−	32	30^a^		28	23^a^			−	−	
	Female		62	61	−	−	−	−	93	67		91	49^a^			−	−	
Rowe et al.	2010	UK	50	82	64.9(9.0)	66.2(7.3)	−	−	16	18	15	22	36	22		0.064	0.371	7
Shao et al.	2005	China	140	144	−	−	80/60	74/70	84	41	15	75	62	7		0.007	0.264	7
Xu et al.	2002	China	144	201	−	−	−	−	80	54	10	105	86	10		0.830	0.149	7
	Early		47	161	−	−	−	−	29	17	1	84	68	9		0.405	0.317	
	Late	China	97	40	−	−	−	−	51	37	9	21	18	1		0.547	0.206	

HWE, Hardy-Weinberg equilibrium, a; GA plus AA.

**Table 2:** Characteristics of the included studies of the association between the COMT Val158Met polymorphism and cognitive dysfunction in PD.

First author	Year	Country	Number of		Mean age	Male/female	PD-NC			PD-CI			HWE		NOS score
			PD-NC	PD-CI			V-V	V-M	M-M	V-V	V-M	M-M	PD-CN	PD-CI	
Dai	2015	China	702	385	63.0	631/456	344	291	67	192	162	33	0.632	0.887	7
Li	2014	China	68	71	59.64(11.61)	78/61	35	28	5	40	24	7	0.852	0.246	7
Wang	2014	China	91	75^a^	66.39	——	52	32	7	34	36	5	0.510	0.264	7
Wang	2014	China	91	99^b^	66.39	——	52	32	7	57	39	3	0.510	0.226	6
Białecka	2012	Poland	29	13	——	——	20	5	4	9	4	0	0.006	0.512	7

PD-NC, PD with no cognitive impairment; PD-CI, cognitive impairment in PD; a, mild cognitive impairment in PD; b, dementia in PD.

**Table 3:** Characteristics of the included studies of the association between the COMT Val158Met and neuro-cognition in PD.

Author	Year	Country	Subjects	Ethnicity	%Male	Age	Met/Met			Val/Met			Val/Val			Phenotype
							M	SD	*n*	M	SD	*n*	M	SD	*n*	
**UPDRS**																
Wu	2012	UK	PD	Caucasians	65	63	25.60	8.50	10	—	—	—	26.80	12.10	10	UPDRS
Xiao	2017	CHINA	PD	Asian	—	58	31.00	11.90	8	22.40	12.10	56	25.80	11.90	79	UPDRS
Morley	2012	USA	PD	Caucasians	74	71	24.00	11.00	56	—	—	—	22.00	11.00	156	UPDRS
Fallon	2015	Netherlands	PD	Caucasians	67	64	31.00	11.00	108	33.00	12.00	184	31.00	9.00	80	UPDRS
Williams-Gray	2007	UK	PD	Caucasians	65	65	26.10	9.40	16	—	—	—	22.50	10.50	16	UPDRS
Zhang	2016	CHINA	PD	Asian	58	60	30.47	12.28	8	30.00	8.75	105	27.26	14.05	137	UPDRS
Williams-Gray	2008	UK	PD	Caucasians	62	64	26.80	10.00	13	—	—	—	23.00	10.80	16	UPDRS
Bäckström	2017	Sweden	PD	Caucasians	59	69	24.94	17.65	42	25.7	12.13	64	30.07	10.19	26	UPDRS
Paul	2016	USA	PD	Caucasians	55	66	20.2	9.3	63	—	—	—	18.8	9.1	168	UPDRS
Hoogland	2010	The Netherlands	PD	Caucasians	54	66	20.00	9.00	37	20	10	84	18.00	9.00	32	UPDRS
**MMSE**																
Wu	2012	UK	PD	Caucasians	65	63	29.60	0.50	10	—	—	—	29.60	0.70	10	MMSE
Fallon	2015	Netherlands	PD	Caucasians	67	64	28.00	2.00	108	29.00	1.00	184	28.00	2.00	80	MMSE
Williams-Gray	2007	UK	PD	Caucasians	65	65	28.70	0.80	16	—	—	—	28.90	1.20	16	MMSE
Zhang	2016	CHINA	PD	Asian	58	60	26.75	3.45	8	26.74	3.50	105	26.93	3.03	137	MMSE
Williams-Gray	2008	UK	PD	Caucasians	62	64	28.80	0.70	13	—	—	—	28.90	1.20	16	MMSE
Bäckström	2017	Sweden	PD	Caucasians	59	69	28.60	1.40	42	28.70	1.40	64	28.70	1.30	26	MMSE
Paul	2016	USA	PD	Caucasians	55	66	28.20	2.60	63	—	—	—	28.30	1.90	168	MMSE
**Combined IQ**																
Wu	2012	UK	PD	Caucasians	65	63	120.20	4.40	10	—	—	—	114.30	8.20	10	NART
Fallon	2015	Netherlands	PD	Caucasians	67	64	102.00	20.00	108	103.00	18.00	184	103.00	18.00	80	NART
Williams-Gray	2007	UK	PD	Caucasians	65	65	114.70	6.20	16	—	—	—	113.10	7.30	16	NART
Zhang	2016	CHINA	PD	Asian	58	60	92.56	16.90	8	97.99	14.94	105	100.88	14.84	137	WAIS-VIQ
Zhang	2016	CHINA	PD	Asian	58	60	83.19	18.39	8	94.98	14.24	105	95.71	12.93	137	WAIS-PIQ
Zhang	2016	CHINA	PD	Asian	58	60	85.24	18.29	8	95.74	14.94	105	97.68	14.16	137	WAIS-FIQ
Williams-Gray	2008	UK	PD	Caucasians	62	64	114.50	6.60	13	—	—	—	114.20	6.90	16	NART
Hoogland	2010	The Netherlands	PD	Caucasians	54	66	105.00	17.00	37	100	18	84	102.00	20.00	32	NART

UPDRS, Unified Parkinson’s Disease Rating Scale; MMSE, Mini Mental State Examination; NART, National Adult Reading Test; WAIS, Wechsler Adult Intelligence Scale; VIQ, Verbal Intelligence Quotient; FIQ, Full Intelligence Quotient; PIQ, Performance Intelligence Quotient.

As shown in **Table 1**, 15 studies were performed on Caucasians. Of the 35 studies, 20 focused only on Asian. Six studies explored the relationships of alleles or genotypes with age at disease onset. Meanwhile, six studies explored the relationships with gender. Nevertheless, three studies were deviated according to HWE of genotype frequencies among the controls.

As shown in **Table 2**, four case-control studies reported the association between COMT polymorphism and cognitive dysfunction in PD, including 890 PD-NC (PD, no cognitive impairment) and 643 PD-CI (PD, cognitive impairment includes mild cognitive impairment and dementia of PD) . Three studies involved the Chinese population. In addition, one was from Poland. The genotype frequencies in all studies fully complied with HWE.

**Table 3** identifies 10 studies included in the meta-analytic to compare cognitive effects between groups of Met homozygotes and Val carriers with PD on cognitive scores. The table includes information about sample characteristics (gender distribution, age, genotype frequencies, ethnicity, etc.) and measures administered.

### Meta-Analysis of the Association Between COMT Val158/108Met and PD Susceptibility

**Figure 2:**
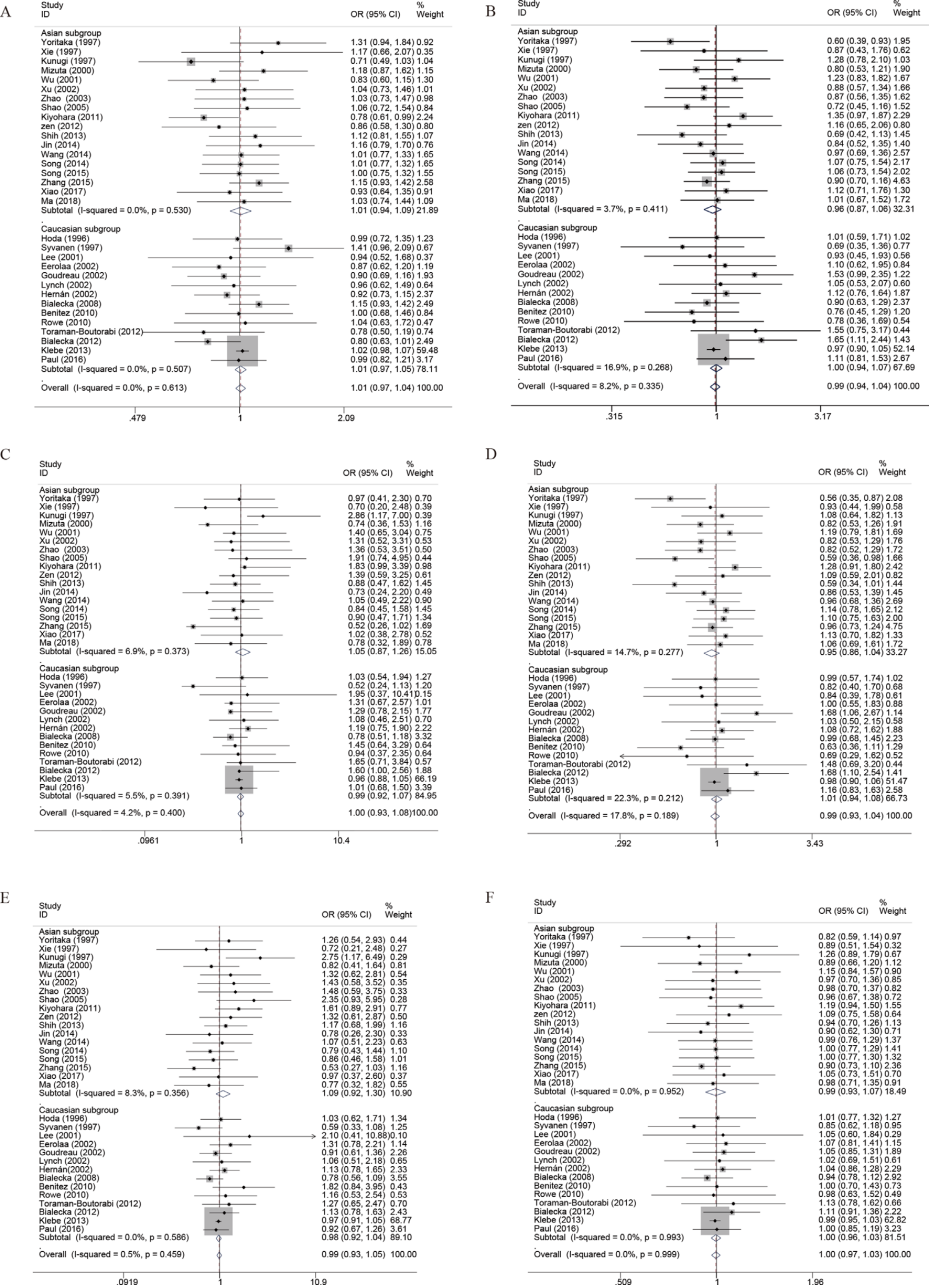
Forest plots for the association between COMT Val158/108Met polymorphism and PD risk. **(A)** Addictive genetic model (A vs G). **(B)** Dominant genetic model (AA +GA vs GG). **(C)** Homozygous model (AA vs GG). **(D)** Heterozygous model (GA vs. GG). **(E)** Recessive genetic model (AA vs. GG + GA). **(F)** Allelic genetic model (A allele distribution frequency of COMT Val158Met gene polymorphism).

Overall, there were 11,428 cases and 16,726 controls included in the analysis. The ORs and 95% CIs in random- and fixed-effect models were calculated according to the values of Q test and I^2^. There was no significant association between COMT Val158/108Met polymorphism and PD susceptibility in the whole population under allelic (OR, 1.01; 95% CI, 0.97–1.04; *P* = 0.667), recessive (OR, 0.99; 95% CI, 0.93–1.05; *P* = 0.792), dominant (OR, 0.99; 95% CI, 0.94–1.04; *P* = 0.671), homozygous (OR, 1.00; 95% CI, 0.93–1.08; *P* = 0.985), heterozygous (OR, 0.99; 95% CI, 0.93–1.04; *P* = 0.655), and additive genetic models (OR, 1.00; 95% CI, 0.97–1.03; *P* = 0.790) (**Figure 2**, **Table 4**). To investigate the exact consequence of the relationship between COMT polymorphism and PD susceptibility, subgroup analyses by ethnicity were performed. No significant association was found in any subgroup under different genetic models between PD risk and COMT genotype (**Figure 2**, **Table 4**).

**Table 4:** Summary of meta-analysis of association between COMT Val158/108Met polymorphism and PD risk.

Genetic model	Pooled OR (95% CI)	Z value	P value	P_Heterogeneity_ (I^2^%)	Publication bias	
					Egger”s test(t)	P value
**Allelic genetic model**						
** Ethnicity**						
Asian subgroup	1.01(0.94,1.09)	0.21	0.833	0.530(0.0%)	0.00	0.998
Caucasian subgroup	1.01(0.97,1.05)	0.38	0.708	0.507(0.0%)	−0.39	0.291
** Gender**						
Male	0.95(0.80,1.11)	0.67	0.502	0.942(0.0%)	−1.12	0.396
Female	0.90(0.75,1.07)	1.25	0.213	0.962(0.0%)	−1.12	0.112
** Onset of PD**						
Early	0.87(0.62,1.23)	0.78	0.436	0.028(63.1%)	0.01	0.998
Late	1.20(1.03,1.40)	2.38	0.017	0.435(0.0%)	0.38	0.792
**Recessive genetic model**						
** Ethnicity**						
Asian subgroup	1.09(0.92,1.30)	0.98	0.326	0.356(8.3%)	0.69	0.564
Caucasian subgroup	0.98(0.92,1.04)	0.63	0.527	0.586(0.0%)	0.36	0.302
** Gender**						
Male	1.04(0.78,1.40)	0.28	0.776	0.778(0.0%)	−2.09	0.076
Female	1.00(0.74,1.36)	0.03	0.980	0.566(0.0%)	1.94	0.107
** Onset of PD**						
Early	1.07(0.75,1.52)	0.36	0.716	0.423(0.0%)	−0.98	0.263
Late	0.71(0.54,0.94)	2.38	0.017	0.457(0.0%)	0.79	0.362
**Dominant genetic model**						
** Ethnicity**						
Asian subgroup	0.96(0.87,1.06)	0.80	0.423	0.411(3.7%)	−0.80	0.483
Caucasian subgroup	1.00(0.94,1.07)	0.03	0.973	0.268(16.9%)	0.41	0.333
** Gender**						
Male	1.07(0.86,1.32)	0.59	0.555	0.979(0.0%)	0.99	0.148
Female	1.21(0.97,1.52)	1.71	0.087	0.865(0.0%)	−0.52	0.613
** Onset of PD**						
Early	1.28(0.98,1.68)	1.79	0.073	0.016(64.0%)	3.04	0.465
Late	0.91(0.74,1.10)	0.99	0.323	0.550(0.0%)	−1.96	0.198
**Homozygous genetic model**						
** Ethnicity**						
Asian subgroup	1.05(0.87,1.26)	0.53	0.594	0.373(6.9%)	0.77	0.554
Caucasian subgroup	0.99(0.92,1.08)	0.21	0.836	0.391(5.5%)	0.56	0.134
** Gender**						
Male	1.07(0.75,1.50)	0.36	0.720	0.822(0.0%)	−0.15	0.926
Female	1.26(0.87,1.81)	1.24	0.216	0.917(0.0%)	0.74	0.455
** Onset of PD**						
Early	1.25(0.82,1.92)	1.04	0.297	0.064(55.1%)	−0.96	0.546
Late	0.73(0.52,1.02)	1.87	0.062	0.493(0.0%)	0.59	0.556
**Heterozygous genetic model**						
** Ethnicity**						
Asian subgroup	0.95(0.86,1.04)	1.11	0.267	0.277(14.7%)	−1.44	0.211
Caucasian subgroup	1.01(0.94,1.08)	0.23	0.819	0.212(22.3%)	0.31	0.477
** Gender**						
Male	1.11(0.86,1.43)	0.81	0.415	0.807(0.0%)	1.77	0.196
Female	1.32(1.00,1.74)	1.93	0.053	0.517(0.0%)	−0.93	0.569
** Onset of PD**						
Early	1.30(0.97,1.75)	1.73	0.084	0.019(66.0%)	5.67	0.329
Late	0.89(0.71,1.13)	0.95	0.343	0.770(0.0%)	−1.58	0.201
**Additive genetic model**						
** Ethnicity**						
Asian subgroup	0.99(0.93,1.07)	0.18	0.861	0.952(0.0%)	−0.05	0.958
Caucasian subgroup	1.00(0.96,1.03)	0.21	0.832	0.993(0.0%)	0.22	0.273
** Gender**						
Male	1.03(0.89,1.19)	0.38	0.702	0.995(0.0%)	0.28	0.713
Female	1.06(0.91,1.24)	0.75	0.453	0.997(0.0%)	0.60	0.150
** Onset of PD**						
Early	1.07(0.90,1.27)	0.77	0.442	0.325(14.0%)	0.03	0.989
Late	0.90(0.79,1.03)	1.50	0.134	0.752(0.0%)	−0.47	0.614

**Figure 3:**
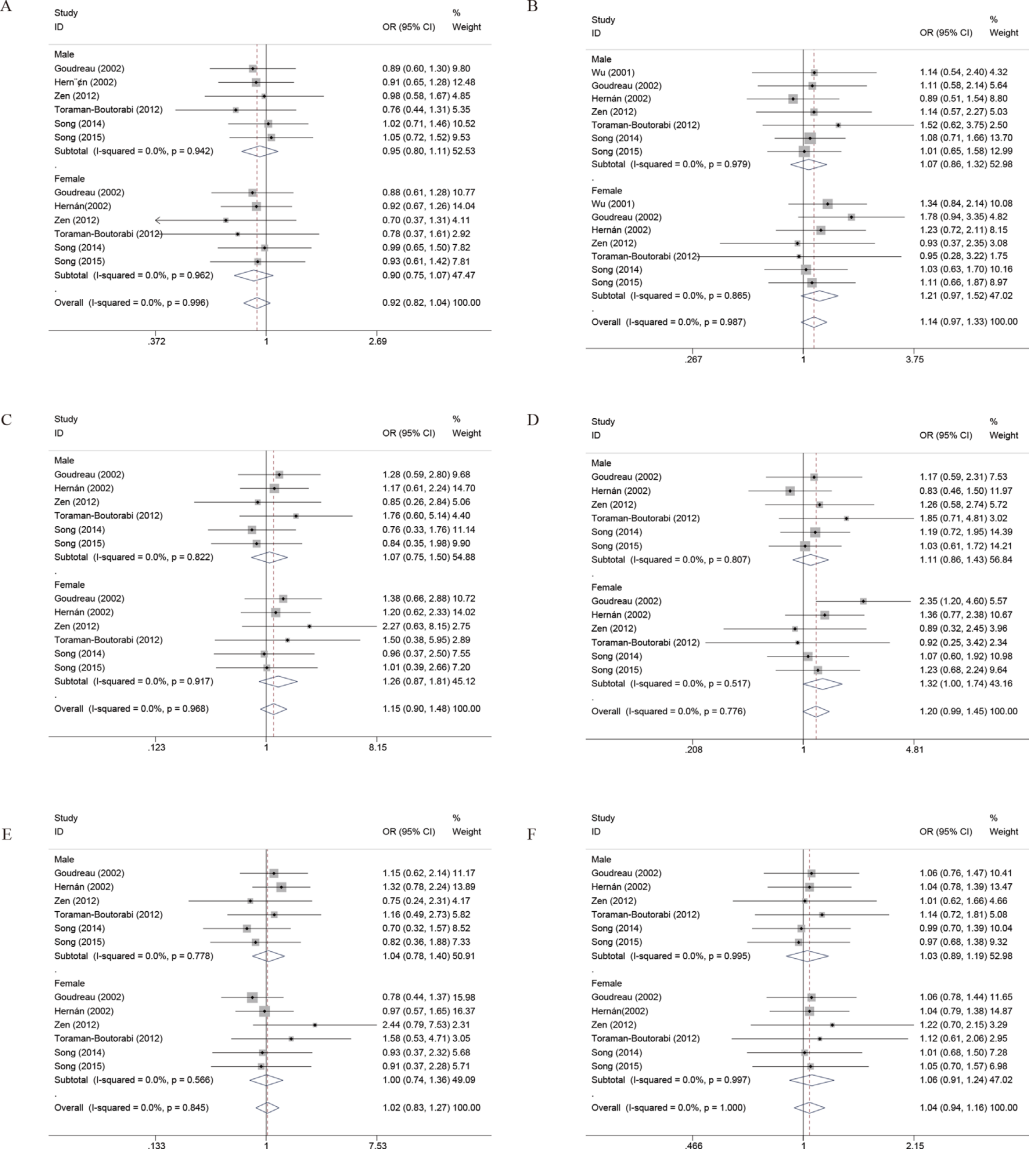
Forest plots for the association between COMT Val158/108Met polymorphism and PD risk-stratified by gender. **(A)** Addictive genetic model (A vs G). **(B)** Dominant genetic model (AA +GA vs GG). **(C)** Homozygous model (AA vs GG). **(D)** Heterozygous model (GA vs. GG). **(E)** Recessive genetic model (AA vs. GG + GA). **(F)** Allelic genetic model (A allele distribution frequency of COMT Val158Met gene polymorphism).

**Figure 4:**
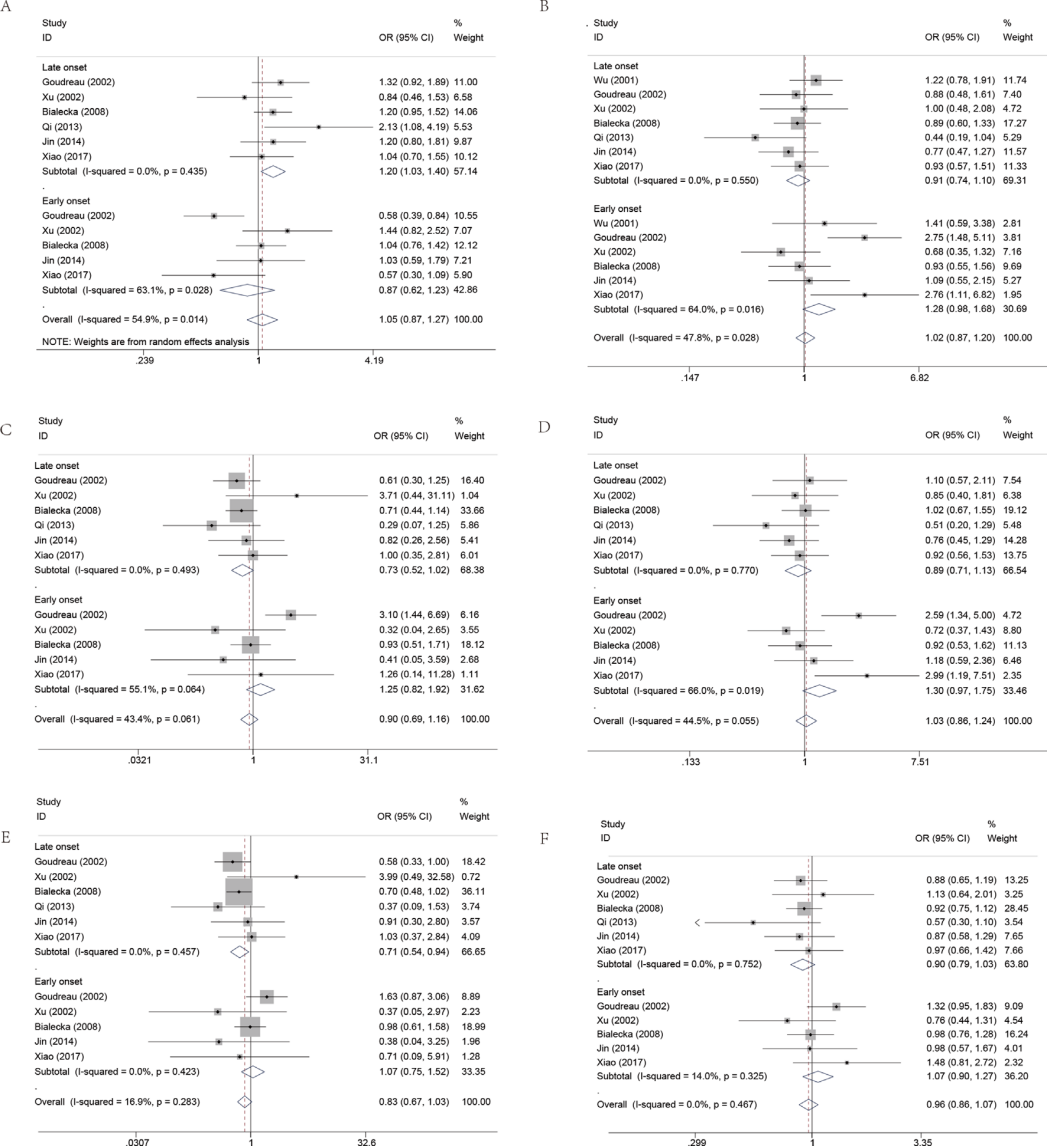
Forest plots for the association between COMT Val158/108Met polymorphism and PD risk-stratified by onset of PD. **(A)** Addictive genetic model (A vs G). **(B)** Dominant genetic model (AA +GA vs GG). **(C)** Homozygous model (AA vs GG). **(D)** Heterozygous model (GA vs. GG). **(E)** Recessive genetic model (AA vs. GG + GA). **(F)** Allelic genetic model (A allele distribution frequency of COMT Val158Met gene polymorphism).

Furthermore, to better explore the role of COMT in PD, we performed the subgroup analysis by the gender and onset of PD. As noted previously, a total of six case-control studies have been reported regarding the association between COMT polymorphism and gender of PD susceptibility. In the gender subgroup, no significant association was detected under all the genetic models, except that a borderline significant association was detected of female PD in the heterozygous genetic models (OR, 1.32; 95% CI, 1.00–1.74; *P* = 0.053) in the pooled populations (**Figure 3**, **Table 4**). In addition, the pooled analyses indicated that there was a signiﬁcant association between COMT polymorphism and late onset of PD susceptibility under recessive (OR, 0.71; 95% CI, 0.54–0.94; *P* = 0.017) and allelic genetic models (OR, 1.20; 95% CI, 1.03–1.40; *P* = 0.017) (**Figure 4**, **Table 4**).

Sensitivity analysis was carried out for this meta-analysis by omitting one study at a time to assess the influence of any single study. When removing one study at the time, there was no significant change in the pooled ORs.

The publication bias of the studies was evaluated by Egger’s test. The results of Egger’s test also suggested that the possibility of publication bias was low (**Table 4**).

### Meta-Analysis of the Association Between COMT Val158/108Met and PD-CI Susceptibility

**Figure 5:**
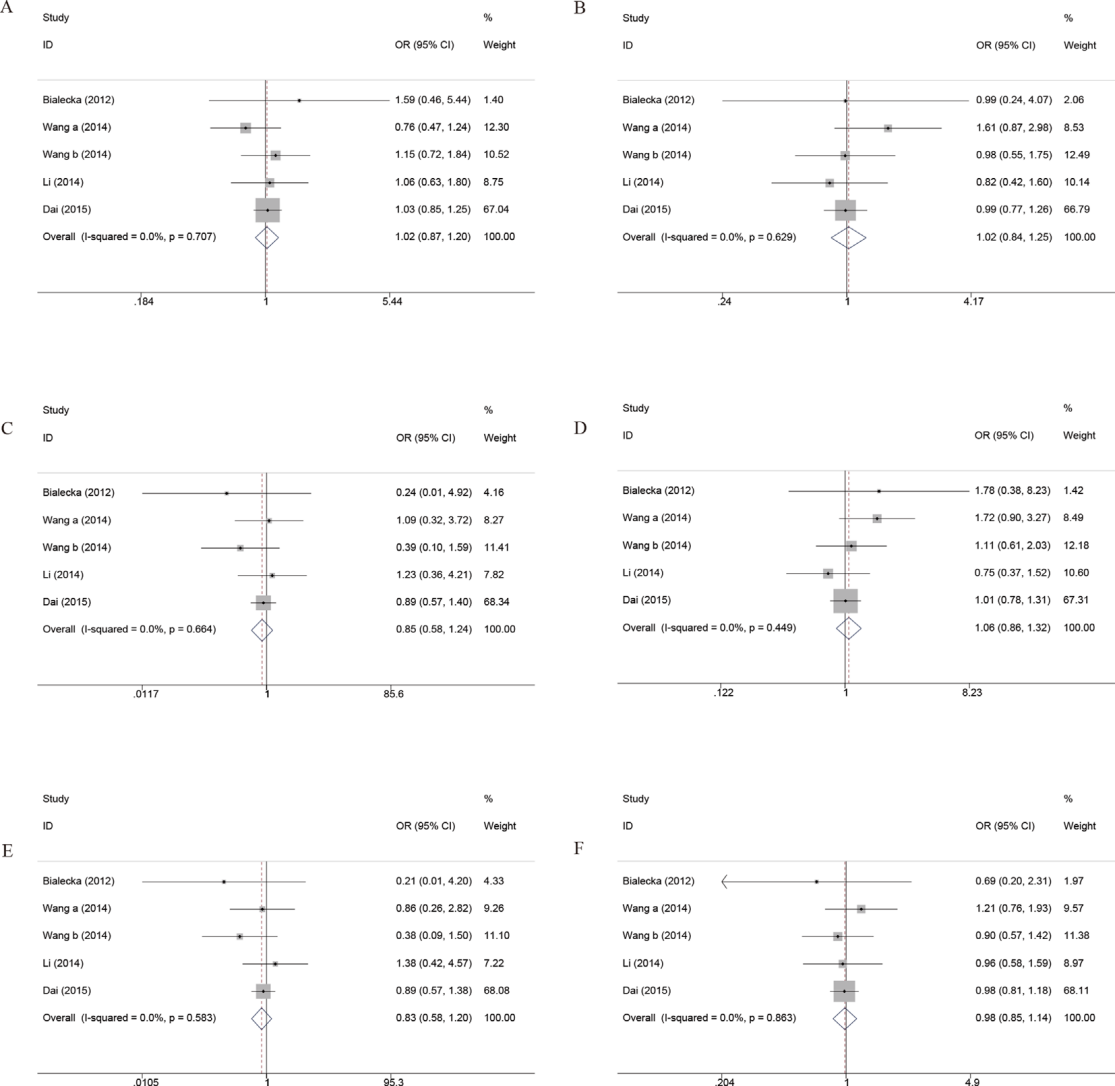
Forest plots for the association between COMT Val158/108Met polymorphism and cognitive impairment in PD. **(A)** Addictive genetic model (A vs G). **(B)** Dominant genetic model (AA +GA vs GG). **(C)** Homozygous model (AA vs GG). **(D)** Heterozygous model (GA vs. GG). **(E)** Recessive genetic model (AA vs. GG + GA). **(F)** Allelic genetic model (A allele distribution frequency of COMT Val158Met gene polymorphism). a, PD-NC vs PD-MCI; b, PD-NC vs PDD.

From the four included studies, we pooled data from 1,533 subjects, including 890 PN-NC and 643 PD-CI. There was no significant association between COMT Val^158/108^Met polymorphism and PD in the whole population under allelic (OR, 1.02; 95% CI, 0.87–1.20; *P* = 0.788), recessive (OR, 0.83; 95% CI, 0.58–1.20; *P* = 0.334), dominant (OR, 1.02; 95% CI, 0.84–1.25; *P* = 0.831), homozygous (OR, 0.85; 95% CI, 0.58–1.24; *P* = 0.402), heterozygous (OR, 1.06; 95% CI, 0.86–1.32; *P* = 0.561), and additive genetic models (OR, 0.98; 95% CI, 0.85–1.14; *P* = 0.836) (**Figure 5**).

Sensitivity analysis was carried out for this meta-analysis by omitting one study at a time to assess the influence of any single study and found no significant change in the pooled ORs. The publication bias of the studies was evaluated by Egger’s test. The results of Egger’s test also suggested that the possibility of publication bias was low (data not shown).

### Meta-Analysis of the Cognitive Effects of the COMT Val158/108Met Polymorphism in PD

#### UPDRS III

**Figure 6:**
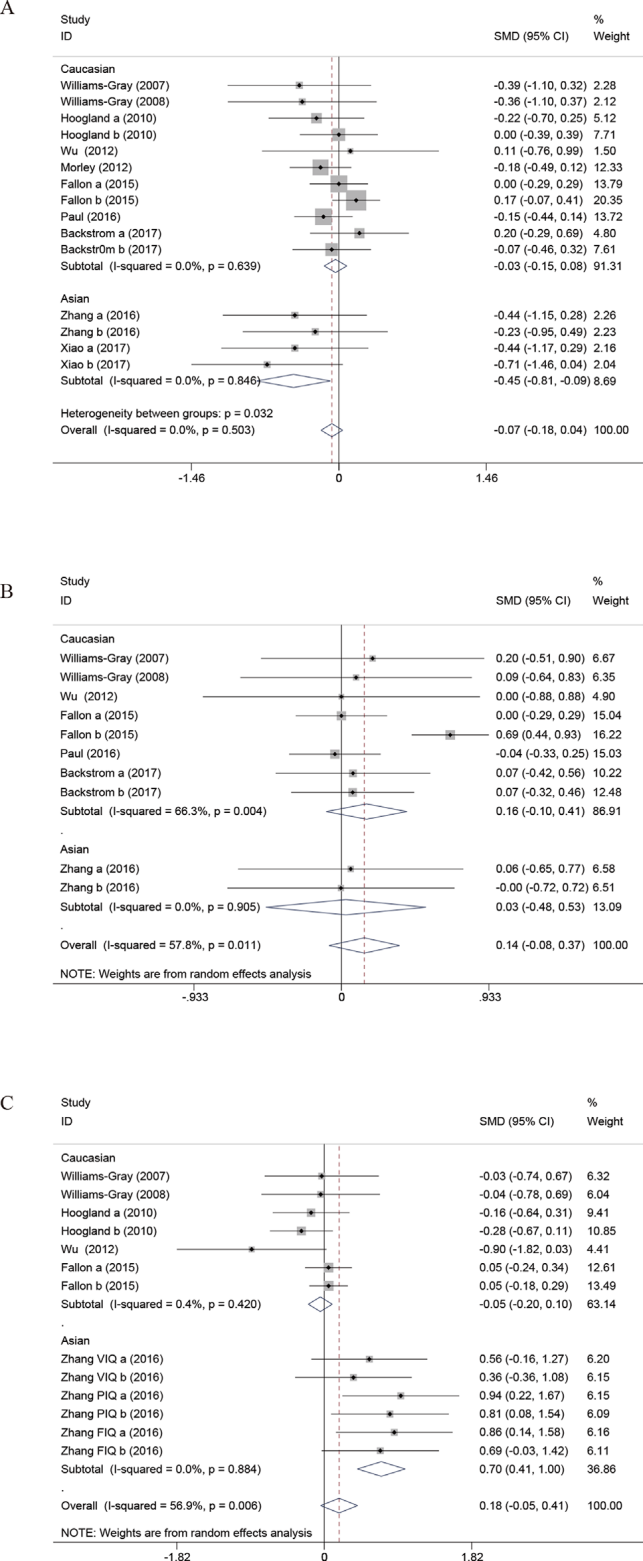
The forest plot for differences between Val carriers and Met homozygotes on the cognitive effects. **(A)** UPDRS III. **(B)** MMSE. **(C)** IQ Score. UPDRS, Unified Parkinson’s Disease Rating Scale; MMSE, Mini Mental State Examination; CI, confidence interval; OR, odds ratio. a, Val/Val vs Met/Met; b, Val/Met vs Met/Met.

Ten independent studies with 1,574 PD patients contributed to the meta-analysis. There was no evidence of between-study heterogeneity (Q-statistic = 13.30; *P* = 0.503; *I* ^2^ = 0%), and random effects analysis indicated no evidence of association (SMD, −0.07; 95% CI, −0.18; 0.04; *P* = 0.206) (**Figure 6A**).

The results of Egger’s test also suggested that the possibility of publication bias was high (*P* = 0.017). However, a subgroup analysis comparing the Asians versus Caucasians was significant, indicating that homozygotes for the Met158 allele have higher UPDRS III scores than the Val allele carriers in Asian population (SMD, −0.45; 95% CI, −0.81 to −0.09; *P* = 0.016). The publication bias of the studies was evaluated by Egger’s test. The results of Egger’s test also suggested that publication bias was low in Asians (*P* = 0.169) or Caucasians (*P* = 0.259).

#### MMSE

Seven independent samples with 835 PD patients contributed to the meta-analysis. There was evidence of between-study heterogeneity (Q-statistic = 21.32; *P* = 0.011; I^2^ = 57.8%), and random effects analysis indicated no evidence of association (SMD, 0.14; 95% CI, −0.08 to 0.37; *P* = 0.203). The results of Egger’s test also suggested that the possibility of publication bias was low (*P* = 0.306). In addition, subgroup analysis by ethnicity indicated that no association was detected in Asian population or Caucasian population (**Figure 6B**).

#### IQ Score

Six independent samples with 856 PD patients contributed to the meta-analysis. Combined IQ score included the National Adult Reading Test (NART) and Wechsler Adult Intelligence Scale (WAIS). There was evidence of between-study heterogeneity (Q statistic = 27.82, *P* = 0.006, *I* ^2^ = 56.9%), and random effects analysis indicated no evidence of association (SMD, 0.18; 95% CI, −0.05 to 0.41; *P* = 0.116).

The results of Egger’s test also suggested that the possibility of publication bias was low (*P* = 0.206), although subgroup analysis by ethnicity detected that homozygotes for the Met158 allele have lower IQ scores than the Val allele carriers in Asian population (SMD, 0.70; 95% CI, 0.41–1.00; *P* < 0.001). Considering that the sample size of Asians is limited, one needs to be cautious of the result and large-scale studies need to clarify the significance of the conclusion (**Figure 6C**).

## Discussion

In the present meta-analysis, we did not find any statistical association between the COMT polymorphism with risks of PD in the overall population, even though five different models were utilized. In the new stratified analyses of population-based studies on different ethnicities (Asians and Caucasians), no association with COMT Val158/108Met polymorphism was shown. Intriguingly, subgroup analyses focusing on gender and onset time demonstrated that COMT Val158/108Met polymorphism has a major impact on the female in the heterozygous genetic model and the late onset in recessive and allelic genetic models. In addition, there was also a faint association between genotype and abstract thought (IQ: WAIS, NART). We also found no evidence of significant publication bias.

For the deep analysis, gender should be considered as a main factor to explore the COMT genotype with cognition in PD. However, not enough data could be obtained for accurate analysis. Sannino et al. (2015, 2017) reported that female COMT Met carriers show reduced cortical thickness after puberty and closely correlated with cognitive functions, which indicates that there is a change of brain structure that can increase the risk of PD. These findings suggest that it is important to appreciate the genetic and gender difference in the patient population. In the context of COMT Val158/108Met polymorphism, female PD patients should be assessed for cognitive function alone in future studies.

Our studies indicated that female carriers of the Met allele of COMT may be at an increased risk for developing PD. The question remains: How could the COMT genotype influence PD development in a gender-specific way? The COMT gene is located on chromosome 22, band q11.2 (Winqvist et al., 1992), and its encoded enzyme degrades a broad group of physiological catechols. This is one of the main mechanisms of terminating DA action in the synapse, together with DA reuptake by the DAT and DA diffusion out of the synapse (Axelrod and Weinshilboum, 1972; Meiser et al., 2013). It is reported that Val is a predominant factor that determines higher COMT activity, which presumably leads to lower synaptic dopamine levels (Chen et al., 2004). The Met/Met genotype causes the change in thermos ability of the enzyme, which is often characterized by decreased enzymatic activity even at 37°C (Boudíková et al., 1990). It may be that women’s hormone levels and thermoregulation differ from men, which illustrates a potential reason why COMT genotype is associated with PD in women alone. Some compelling evidence also demonstrated that estrogens may modulate COMT gene expression and protein activity *via* estrogen receptor function (Schendzielorz et al., 2011). As mentioned above, there is another possibility that female COMT Met carriers may remodel brain structures involved in the neurobiology of PD (Sannino et al., 2015).

Importantly, it was also reported that the Val allele had a significant effect on the age of onset of PD (Jimenez-Jimenez et al., 2014). This genotype may confer a greater risk of PD because of higher catecholaminergic metabolism, leading to an accelerated use of endogenous dopamine reservoirs as well as activating catabolism of COMT substrates (Bialecka et al., 2008). In our analysis, the result was consistent with the previous literature. Carriers of the Val allele of COMT Val158/108Met polymorphism may be at an increased risk for late-onset PD.

Cognitive disorders associated with PD could be so mild that they are under-recognized and do not alter daily living activities (Papagno and Trojano, 2018). A wealth of evidence has shown that the circuitry of dorsolateral prefrontal cortex and the front striatal (Beyer et al., 2007) supports various advanced brain functions (Arnsten, 1998). It has been also shown that regulating the level of DA can improve the specific cognitive function relying on this circuitry (Lewis et al., 2005). Watanabe et al. (1997) also identified that the DA level in prefrontal cortex was strongly related to the performance of working memory. Yerkes–Dodson type U-shape relationship governs the DA modulation of neural function, which means reduction or excessive increases of DA will selectively impair specific cognitive function (Rowe et al., 2008). Further evidence demonstrating the role of DA on cognitive function in PD has been obtained from the study of polymorphisms of COMT, which regulates prefrontal cortical DA turnover, as well as activation in prefrontal–striatal regions (Nombela et al., 2014).

**Figure 7:**
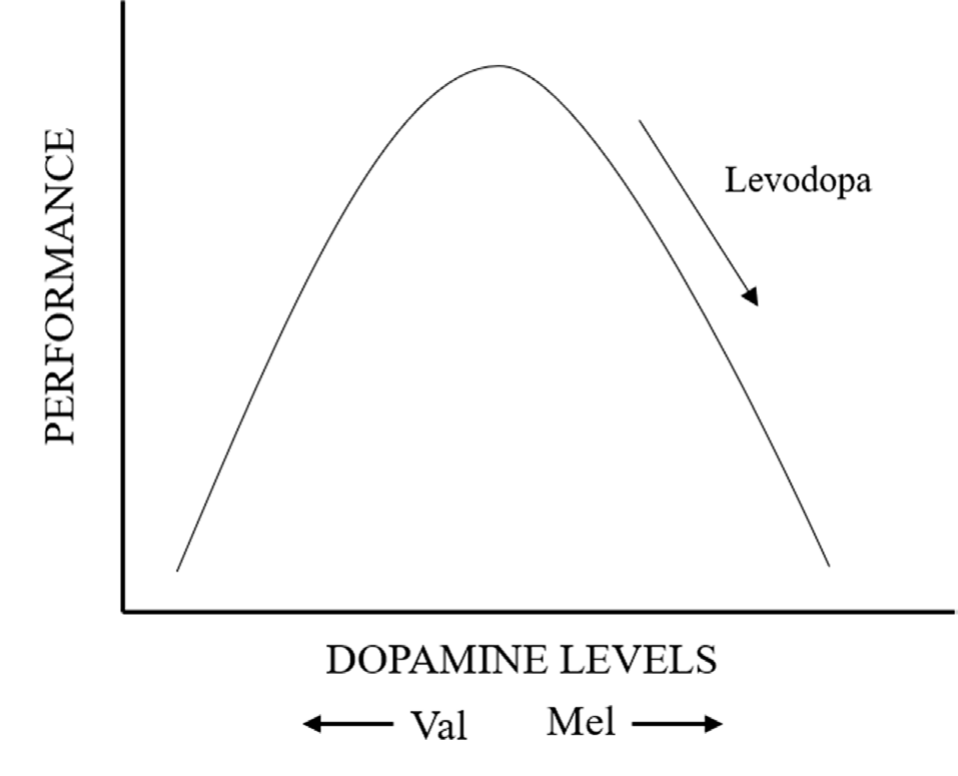
Diagram of inverted U-shaped relationship between prefrontal function and dopamine levels.

In our analysis of association between cognitive and motor abilities in PD and COMT Val158/108Met polymorphism, there is no positive evidence. During the seriatim heterogeneity test and sensitivity analysis, we found one study that if we removed from the analysis, the results would have been reversed. The eccentric study was about the European population. First and foremost, we made a subgroup analysis of ethnics. The result showed that the relationship between COMT and UPDRS III, IQ was appreciable in Asian, but not Europeans. Asians with Met carrier conferred an increased risk of high UPDRS III score, meaning a more severe motor impairment. Meanwhile, Asian patients with Met/Met genotype performed significantly worse on abstract thought (IQ: WAIS, NART). After further analysis on these two studies, we found that the levodopa dose was lower in subjects with Met/Met genotype than Val/Val group. However, in other studies, almost all PD patients with Met/Met genotype took more LODA. Given the change in the results before and after inclusion, LODA dose was a major factor and concern. It was reported that the relationship between prefrontal function and dopamine levels follows an inverted U-shaped curve (**Figure 7**) (Williams-Gray et al., 2007). According to this theory, we speculated that, among PD patients, those with low activity COMT (Met/Met alleles) who take more LODA have worse performance in motor and cognition because of excessive DA levels. This is believed to contribute to impaired cognitive performance in prefrontal regions, which explains the reason of disruptive changes when one study was omitted. To sum up, factors about dosage and type of anti-PD medication should account for many reasons. Dopamine supplements may mask some linking of COMT polymorphisms and cognitive function. Studies have confirmed that high doses of dopamine reduce the expression of dopamine transporters in the prefrontal cortex, which could lead to cumulative toxicity of dopamine and make patients appear to have cognitive impairment like the patients with low-activity COMT. In other words, if PD patients with low-activity COMT use large-dose levodopa for a long time, cognitive impairment can be more severe. However, even if the use and types of anti-PD drugs have been taken into account, we could not complete this analysis due to the uncertainty and ambiguity of information.

The Val158/108Met polymorphism remains a plausible candidate that may contribute to cognitive function deficits in PD. Although the results do not meet our expectations, it does not negate the need for scientific rigor in both the analysis and the reporting of results. At the very least, we cannot exclude the possibility that COMT genotype has a small influence on cognitive phenotypes and PD susceptibility in women. These results are partly in agreement with the previous meta-analysis, which reported that there is a significant association between COMT rs4680 and the risk for PD in the total series, as well as homozygosity for the low-activity allele in the Asiatic population (Jimenez-Jimenez et al., 2014). In addition, our analysis also further revealed the relationship between COMT genotype and PD of female and late onset. Meanwhile, in the aspect of cognition of PD, it also gives some risk analysis and discussion.

There are several potential limitations in the present meta-analysis. First, there is marginal significance among female PD patients, which encourages us; further research on the association of gender of PD and COMT Val158/108Met polymorphism should be carried out. Second, sample size limited our ability to investigate the accuracy of association of COMT Val158/108Met polymorphism and cognitive dysfunctions in PD. Additionally, limited data were obtained to explore the COMT genotype and cognition in early or late onset of PD. Due to the uneven nature of population genotype frequencies in different ethnic groups, the association of COMT Val158/108Met polymorphism and cognitive dysfunctions in PD needs further confirmation. The cognition scale indicators were multitudinous, and we were underpowered to test the combined effect in the current sample. Lastly, more studies are desirable in COMT polymorphism studies; also, the type and dose of anti-PD drug should be included specifically.

Our findings highlight the impact that COMT genetic factors can have on the susceptibility of PD and cognitive performance, specifically differences in female, late-onset PD patients, and IQ.

## Conclusion

In summary, the results of our meta-analysis indicated that the COMT Val158/108Met polymorphism may be associated with the risk for PD in female or late-onset PD. Meanwhile, Met/Met carriers of Asian population performed significantly worse than the Val allele carriers in IQ score and UPDRS III. Considering the above potential limitation, these conclusions need to be further confirmed in future research.

## Author Contributions

All authors listed have made substantial, direct, and intellectual contribution to the work and approved it for publication.

## Conflict of Interest Statement

The authors declare that the research was conducted in the absence of any commercial or financial relationships that could be construed as a potential conflict of interest.
